# Interfacial Chemical Selection via Post-Silanization Processing Governs Dispersion Stability of 3Y-TZP Nanoparticles: A Qualitative Assessment of Interfacial Characteristics

**DOI:** 10.3390/polym18091089

**Published:** 2026-04-29

**Authors:** Tunyaporn Parmornsupornvichit, Awutsadaporn Katheng, Watcharapong Tonprasong, Paweena Kongkon

**Affiliations:** Department of Restorative Dentistry, Faculty of Dentistry, Naresuan University, 99 Moo 9, Mueang Phitsanulok, Phitsanulok 65000, Thailand; tunyapornp67@nu.ac.th (T.P.); watcharapongt@nu.ac.th (W.T.); paweenako@nu.ac.th (P.K.)

**Keywords:** 3Y-TZP nanoparticles, silanization, post-silanization processing, dispersion stability, surface chemistry

## Abstract

This study investigated the effect of post-silanization processing on the surface chemistry and dispersion stability of 3 mol% yttria-stabilized tetragonal zirconia polycrystal (3Y-TZP) nanoparticles intended for the reinforcement of dental photopolymer resins. The nanoparticles were silanized using 3-Methacryloxypropyltrimethoxysilane and subjected to different post-treatment protocols, including control, drying, and centrifugation. Particle morphology was examined using field-emission scanning electron microscopy (FE-SEM) and transmission electron microscopy (TEM). Dispersion behavior was analyzed by dynamic light scattering (DLS) and zeta potential measurements, performed in triplicate (*n* = 3), while surface chemical modifications were evaluated using Fourier transform infrared spectroscopy (FT-IR) and X-ray photoelectron spectroscopy (XPS). Post-silanization processing significantly influenced nanoparticle surface chemistry and dispersion stability. Centrifugation promoted the formation of Si–O–Zr and Si–O–Si linkages, reduced loosely adsorbed silane species, decreased particle agglomeration, and increased zeta potential magnitude, resulting in a more uniform hydrodynamic size distribution compared to the dried group (Z-average ≈ 814 nm, PDI ≈ 0.44). These findings suggest that post-silanization centrifugation acts as an interfacial selection mechanism that distinguishes covalently grafted silane from weakly adsorbed species. Within the limitations of this in vitro study, further investigations under varied conditions are required to confirm broader applicability.

## 1. Introduction

Additive manufacturing has been increasingly adopted in dentistry for the fabrication of provisional restorations due to its advantages in digital workflow integration, design flexibility, and manufacturing efficiency. Among additive technologies, vat-photopolymerization systems such as digital light processing (DLP) and stereolithography (SLA) are widely used for producing provisional dental restorations with acceptable esthetics and dimensional accuracy. However, despite these advantages, 3D-printed dental photopolymer resins generally exhibit inferior mechanical durability compared with conventional and CAD/CAM-milled polymethyl methacrylate (PMMA) materials, particularly under long-term functional loading and aging conditions [1,2,3,4]. This limitation has restricted the broader clinical application of additively manufactured provisional restorations in load-bearing situations.

To overcome these material limitations, nanoparticle reinforcement has emerged as an effective strategy to enhance the performance of polymer-based dental materials. The incorporation of inorganic nanoparticles into polymer matrices has been shown to improve mechanical strength, wear resistance, and structural stability by promoting stress transfer and inhibiting crack propagation [5,6,7]. Among various nanofillers investigated for dental applications, zirconia nanoparticles have attracted considerable attention due to their favorable mechanical properties, chemical stability, and biocompatibility [8,9,10].

In particular, 3 mol% yttria-stabilized tetragonal zirconia polycrystals (3Y-TZP) present several advantages at the nanoscale. The tetragonal phase of zirconia is stabilized at room temperature by yttria addition, enabling transformation toughening and high fracture resistance [11,12]. At the nanoscale, 3Y-TZP particles exhibit increased surface energy and enhanced chemical reactivity, which may promote stronger interfacial interactions with polymer matrices while maintaining phase stability [13,14,15]. These characteristics make 3Y-TZP nanoparticles promising candidates for reinforcing dental photopolymer resins.

Nevertheless, the effectiveness of nanoparticle reinforcement depends not only on the intrinsic properties of the filler but also on the quality of interfacial bonding between the inorganic nanoparticles and the organic polymer matrix. Without adequate interfacial coupling, nanoparticles may behave merely as inert fillers, leading to agglomeration, poor stress transfer, and compromised material performance [16,17]. To address this issue, silanization is commonly employed as a surface modification technique to chemically bridge inorganic fillers and organic polymers. Silane coupling agents, such as methacrylate-functional silanes, can form covalent bonds with hydroxyl groups on zirconia surfaces while simultaneously co-polymerizing with methacrylate-based resins, thereby enhancing filler–matrix compatibility [18,19,20].

While numerous studies have reported the benefits of silanized zirconia nanoparticles in dental resin systems, the majority of the existing literature focuses on nanoparticle concentration and resultant mechanical or optical properties, with limited attention to the physicochemical aspects of nanoparticle surface preparation. In particular, post-silanization processing steps, such as drying, washing, or separation techniques, are often briefly mentioned or omitted entirely, despite their potential influence on surface chemistry and particle agglomeration [21,22,23]. Improper post-silanization treatment may lead to the presence of loosely bound or unreacted silane species, which can promote nanoparticle aggregation and reduce dispersion stability.

The influence of post-silanization processing methods, such as drying versus centrifugation, on the surface chemistry and dispersion behavior of silanized 3Y-TZP nanoparticles remains insufficiently investigated. Understanding how these processing parameters affect silane bonding efficiency, surface charge, and agglomeration behavior is essential for establishing a mechanistic foundation for the design of dental nanocomposite photopolymer resins. Therefore, the objective of this study was to investigate the effect of post-silanization processing on the surface chemistry and dispersion stability of 3Y-TZP nanoparticles. The null hypothesis was that post-silanization processing would not significantly influence nanoparticle surface chemistry or dispersion characteristics. The alternative hypothesis was that processing-dependent surface chemistry governs nanoparticle dispersion behavior, thereby providing a critical physicochemical basis for understanding the interfacial selection mechanism. While clinical-level mechanical assessments are beyond the scope of this fundamental study, the insights gained into nanoparticle dispersion and surface chemistry are essential prerequisites for the future development of high-performance dental photopolymer resins.

## 2. Materials and Methods

Commercial yttria-stabilized zirconia nanoparticles (3Y-TZP; TZ-3Y-E, Tosoh Corporation, Tokyo, Japan) were used as inorganic fillers. According to the manufacturer, the nanoparticles have a specific surface area of 14.9 m^2^ g^−1^ and an average crystallite size of approximately 27 nm. 3-Methacryloxypropyltrimethoxysilane (MPS; Silane A-174, Sigma-Aldrich, St. Louis, MO, USA) was used as the silane coupling agent. Ethanol (absolute, 99.7%, AR1380-P, RCI Labscan, Bangkok, Thailand) and isopropanol (2-propanol, AR1162-P, RCI Labscan, Bangkok, Thailand) were used as solvents for silanization and post-silanization treatments.

Prior to silanization, 3Y-TZP nanoparticles were dried in a hot-air oven at 100 °C for 16 h to remove adsorbed moisture and improve surface hydroxyl reactivity. The silane solution was prepared by dissolving 3-Methacryloxypropyltrimethoxysilane (MPS) in 80 vol% ethanol with magnetic stirring at room temperature for 1 h to allow hydrolysis of methoxy groups.

The required amount of silane was calculated based on the specific surface area of the nanoparticles using the modified Posthumus equation [24], assuming monolayer surface coverage. The silane mass (X, g) was calculated as

X = m_3Y-TZP_ × MW_MPS_ × M_MPS_ × BET_3Y-TZP_ × 10^−6^

where m_3Y-TZP_ is the mass of nanoparticles (g), MW_MPS_ is the molecular weight of MPS (248.35 g mol^−1^), M_MPS_ is the assumed surface coverage (6.9 µmol m^−2^), and BET_3Y-TZP_ is the specific surface area of the nanoparticles (14.9 m^2^ g^−1^). The calculated silane mass was converted to volume using the density of MPS (*p* = 1.045 g mL^−1^ at 25 °C). Based on this calculation, 0.0255 g (0.024 mL) of MPS was required per gram of 3Y-TZP nanoparticles.

The dried nanoparticles were introduced into the hydrolyzed silane solution and stirred at 200 rpm at room temperature for 2 h. The suspension was subsequently refluxed at 70 °C for 4 h to allow silane condensation on the nanoparticle surface.

Post-silanization processing was performed based on a previously described purification approach [24], with minor modifications. After silanization, the suspension was cooled to room temperature and divided into two portions. One portion was directly dried in a hot-air oven at 50 ± 5 °C for 16 h to obtain dried silanized nanoparticles.

The remaining portion was diluted with isopropanol (99 vol%) at a 3:1 (*v*/*v*) ratio and magnetically stirred for 10 min prior to centrifugation at 4500 rpm for 1 h to remove unreacted and self-condensed silane species. The supernatant was discarded, and the sediment was redispersed in fresh isopropanol and centrifuged twice (4500 rpm, 15 min each). The purified sediment was subsequently dried at 50 ± 5 °C for 16 h.

Fourier transform infrared spectroscopy (FT-IR; INVENIO S, Bruker, Billerica, MA, USA) was performed to identify silane-related functional groups. After post-silanization processing, the dried nanoparticles were gently ground using an agate mortar and pestle to obtain a homogeneous powder. Approximately 1 g of the powdered sample was mixed with spectroscopic-grade KBr and compressed into transparent pellets using a hydraulic press. The pellets were immediately analyzed to minimize moisture adsorption. Spectra were recorded over the range of 400–4000 cm^−1^ at a resolution of 4 cm^−1^ with 32 scans under nitrogen purge conditions.

X-ray photoelectron spectroscopy (XPS; PHI VersaProbe 4, ULVAC-PHI, Chigasaki, Japan) using monochromatic Al Kα radiation (1486.6 eV) was performed on control and centrifuged samples. Powdered specimens were mounted on carbon tape without conductive coating and analyzed under ultra-high vacuum. Charge neutralization was applied during acquisition, and all spectra were calibrated using the C 1s peak at 284.8 eV as reference. Survey spectra were first obtained to determine elemental composition, followed by high-resolution spectra of Si 2p, Zr 3d, O 1s, and C 1s. Peak deconvolution was performed using a Gaussian–Lorentzian mixed line shape after Shirley background subtraction. The Si 2p spectra were deconvoluted to distinguish siloxane (Si–O–Si) and zirconia-bonded silane (Si–O–Zr) components. The O 1s spectra were analyzed to differentiate the lattice oxygen of zirconia and silane-derived oxygen species, while the Zr 3d peaks served as the substrate reference for evaluating surface coverage. The relative atomic concentrations were calculated to assess silane coupling efficiency.

Prior to morphological and dispersion analyses, the nanoparticles were gently ground using an agate mortar and pestle to obtain a homogeneous powder.

For transmission electron microscopy (TEM), a small amount of powder was ultrasonically dispersed in ethanol, and a drop of the suspension was deposited onto a carbon-coated copper grid and dried at room temperature. The morphology and agglomeration of the nanoparticles were examined using a TEM (Talos F200X Gen2, Thermo Fisher Scientific, Waltham, MA, USA) operated at an accelerating voltage of 80 kV for both control and centrifuged samples, representing the baseline and optimized dispersion conditions, respectively.

Surface morphology was further evaluated using field-emission scanning electron microscopy (FE-SEM; JSM-IT800SHL, JEOL, Tokyo, Japan). The powders were mounted on carbon tape attached to aluminum stubs and observed without conductive coating under high vacuum at an accelerating voltage of 5–10 kV.

Particle size distribution and zeta potential were measured using a Zetasizer Nano ZSP (Malvern Instruments, Malvern, UK). The nanoparticles were dispersed in ethanol and ultrasonicated prior to measurement. All measurements were performed at 25 °C in triplicate to evaluate dispersion behavior and colloidal stability.

Comparative analyses were performed among the control, dried, and centrifuged groups to evaluate the effects of post-silanization processing. For mechanistic evaluation, XPS was selectively performed on the control and centrifuged groups to represent baseline and optimized interfacial states, allowing mechanistic comparison between non-treated and chemically stabilized conditions.

Data for DLS (Z-average and PDI) and zeta potential measurements were expressed as mean ± standard deviation (SD) from triplicate analyses (*n* = 3). Statistical significance was determined using one-way analysis of variance (ANOVA) followed by Tukey’s post hoc test, with a *p*-value < 0.05 considered statistically significant. For FT-IR, XPS, SEM, and TEM, representative data from single measurements were presented to characterize the surface and morphological properties.

## 3. Results

### 3.1. FT-IR Analysis of Surface Functional Groups

**Figure 1:**
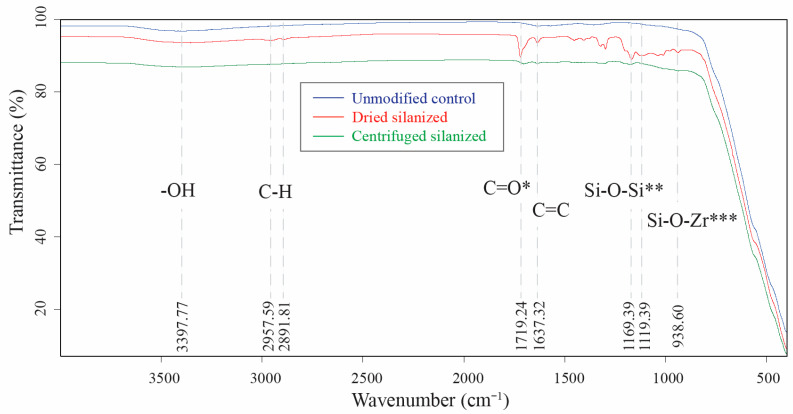
FT-IR spectra of unmodified (control), dried silanized, and centrifuged silanized 3Y-TZP nanoparticles. The control sample exhibited a broad O–H stretching band around 3400 cm^−1^. Following silanization, additional bands corresponding to methacrylate-functional silane were observed, including C–H stretching (2950–2850 cm^−1^), C=O stretching (~1720 cm^−1^), and C=C vibrations (1630–1650 cm^−1^). Bands in the 1100–1000 cm^−1^ region, assigned to Si–O–Si and Si–O–Zr linkages, confirmed the presence of silane-derived surface species. * C=O stretching (methacrylate group), indicating the presence of organic silane moieties; ** Si–O–Si stretching (siloxane network), associated with self-condensation of silane molecules; *** Si–O–Zr stretching, indicative of covalent bonding between silane and the zirconia surface.

Figure 1 presents the FT-IR spectra of unmodified (control), dried silanized, and centrifuged silanized 3Y-TZP nanoparticles. The control sample exhibited a broad absorption band centered around 3400 cm^−1^, corresponding to O–H stretching vibrations of surface hydroxyl groups, along with characteristic Zr–O stretching bands in the 500–450 cm^−1^ region.

The FTIR spectra of silanized nanoparticles revealed the emergence of characteristic organic and siloxane-related bands following surface modification. A distinct absorption band at approximately 1719 cm^−1^, assigned to the C=O stretching vibration of the methacrylate group, was more pronounced in the dried silanized group compared to the centrifuged counterpart. Similarly, the Si–O–Si band observed in the region of 1100–1130 cm^−1^ exhibited higher intensity in the dried group. The enhanced intensity of both C=O and Si–O–Si bands in the dried samples suggests the presence of excess and loosely adsorbed silane species, as well as the formation of self-condensed siloxane networks. In contrast, the reduced intensity of these bands in the centrifuged group indicates the effective removal of non-covalently bound and oligomeric silane species, resulting in a more controlled surface modification.

Overall, the spectral features indicate that the zirconia framework remained chemically stable after treatment, while the emergence of organic-related bands confirms successful silane grafting onto the nanoparticle surface. Variations in peak intensity between the two silanized groups suggest differences in surface coverage, likely influenced by the post-silanization purification process.

### 3.2. Morphological Characterization (FE-SEM and TEM)

**Figure 2:**
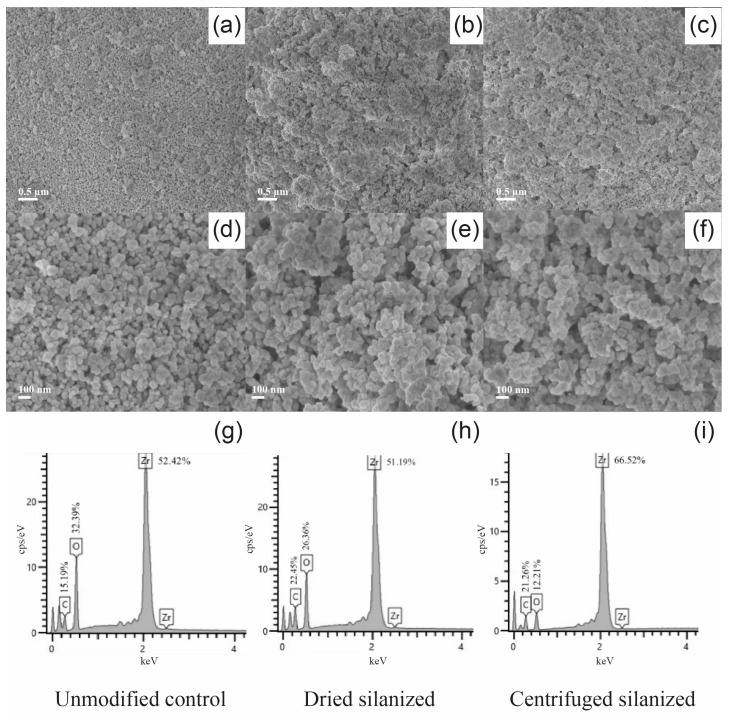
FE-SEM micrographs and corresponding EDS spectra of 3Y-TZP nanoparticles after post-silanization processing: (**a**,**d**,**g**) unmodified control; (**b**,**e**,**h**) dried silanized; and (**c**,**f**,**i**) centrifuged silanized. FE-SEM images were acquired at magnifications of 15,000× (**a**–**c**) and 50,000× (**d**–**f**), respectively. Corresponding EDS spectra are presented in (**g**–**i**).

Representative FE-SEM micrographs and EDS spectra of 3Y-TZP nanoparticles subjected to different post-silanization processing conditions are shown in Figure 2.

At higher magnification (15,000×), structural differences between the treated groups became apparent. The dried silanized sample displayed irregular and heterogeneous aggregates with rough surface texture (Figure 2b), suggesting particle clustering induced by solvent evaporation. In contrast, the centrifuged sample exhibited smaller and more uniformly distributed aggregates (Figure 2c).

At 50,000× magnification, the dried sample showed partially fused particle boundaries and indistinct separation (Figure 2e), whereas the centrifuged sample revealed more discernible individual nanoparticles with well-defined interparticle boundaries and lower aggregate compactness (Figure 2f).

EDS spectra (Figure 2g–i) confirmed zirconium (Zr) and oxygen (O) as the dominant elements in all samples, consistent with the zirconia composition. Carbon (C) signals were also detected, which can be attributed to surface contamination and/or the presence of organic silane species. Silicon (Si) was not clearly resolved in the silanized groups, likely due to the limited sensitivity of EDS for detecting ultrathin organosilane layers. Therefore, complementary surface-sensitive techniques, such as FT-IR and XPS, were employed to further elucidate the chemical modification and interfacial bonding.

**Figure 3:**
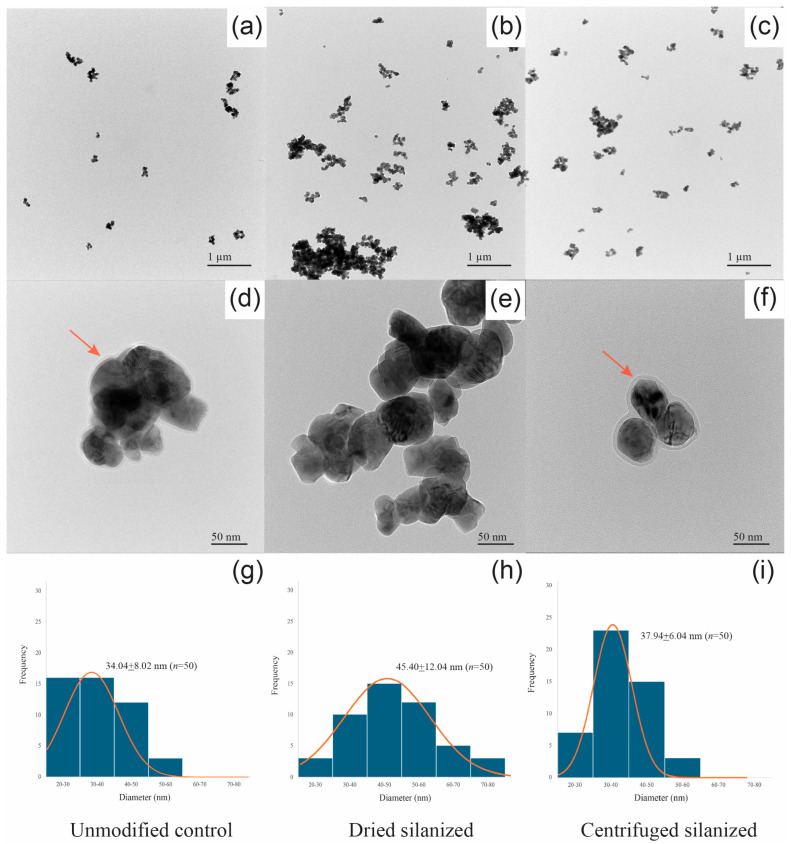
TEM micrographs and particle size distributions of 3Y-TZP nanoparticles under different post-silanization conditions: (**a**,**d**) unmodified control, (**b**,**e**) dried silanized, and (**c**,**f**) centrifuged silanized, acquired at low and high magnifications, respectively. Corresponding particle size distributions derived from TEM images are shown in (**g**–**i**). Red arrows indicate representative individual nanoparticles or interparticle boundaries. The orange curves represent the fitted Gaussian distribution of particle size.

TEM observation (Figure 3) revealed nanoscale differences in particle organization among the unmodified control, dried silanized, and centrifuged silanized samples. The control sample (Figure 3a,d) showed aggregated nanoparticles with indistinct interparticle boundaries. The dried silanized sample (Figure 3b,e) exhibited the most densely packed structure, with tightly clustered nanoparticles and minimal interparticle spacing, likely due to drying-induced aggregation during solvent evaporation. In contrast, the centrifuged silanized sample (Figure 3c,f) demonstrated improved dispersion, characterized by more separated nanoparticles with clearer outlines and reduced interparticle contact. A halo-like interfacial contrast was observed around individual particles in the control and centrifuged silanized samples (Figure 3d,f), whereas this feature was not clearly evident in the dried silanized sample. The contrast appeared more continuous and better defined in the centrifuged silanized sample, suggesting a more uniform interfacial structure following post-silanization processing.

Particle size distributions (Figure 3g–i) further supported these observations. The dried silanized sample exhibited a broader distribution with larger apparent particle sizes (45.40 ± 12.04 nm), consistent with aggregation, whereas the centrifuged silanized sample showed a narrower distribution and smaller average particle size (37.94 ± 6.04 nm). The control sample presented an intermediate particle size (34.04 ± 8.02 nm).

### 3.3. Dispersion Behavior and Colloidal Stability (DLS and Zeta Potential)

**Figure 4:**
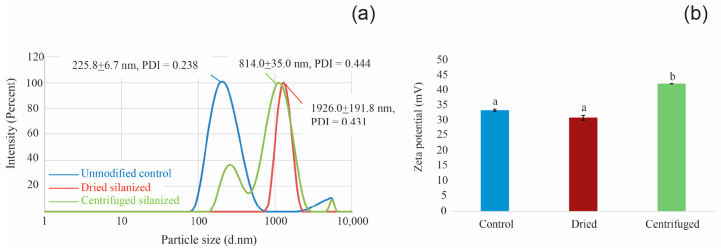
Dynamic light scattering (DLS) size distributions and zeta potential measurements of 3Y-TZP nanoparticle suspensions under different post-silanization conditions: unmodified control, dried silanized, and centrifuged silanized. (**a**) Intensity-weighted hydrodynamic size distributions showing a narrow peak for the control and broadened, right-shifted distributions for the dried and centrifuged samples. (**b**) Zeta potential values of the corresponding suspensions. Different letters indicate statistically significant differences among groups (*p* < 0.05).

The DLS and zeta potential results (Table 1), together with the corresponding distributions shown in Figure 4, demonstrate distinct differences in dispersion behavior among the tested groups.

The unmodified control exhibited the smallest hydrodynamic diameter (Z-average: 225.8 ± 6.7 nm) with a relatively narrow size distribution (PDI: 0.238 ± 0.012), as also reflected by the sharp and narrow peak in Figure 4a. This apparent reduction does not reflect true dispersion but rather compact aggregation of unmodified nanoparticles behaving as single scattering entities. In contrast, the dried silanized sample showed a markedly increased hydrodynamic diameter (1926.0 ± 191.8 nm) and broader distribution (PDI: 0.431 ± 0.047), consistent with the right-shifted and broadened peak observed in Figure 4a. These results indicate severe aggregation induced by the drying process. The centrifuged sample exhibited a reduced hydrodynamic size (814.0 ± 35.0 nm) relative to the dried group, with a distribution peak shifted toward smaller sizes (Figure 4a). Although the PDI remained relatively high (0.444 ± 0.045), the reduction in large-size populations suggests partial improvement in dispersion following post-silanization purification.

Zeta potential measurements (Figure 4b) showed positive surface charges for all groups. The control and dried samples exhibited comparable zeta potentials (+33.4 ± 0.4 mV and +31.0 ± 0.8 mV, respectively), whereas the centrifuged sample showed a significantly higher value (+42.3 ± 0.1 mV). This increase indicates enhanced electrostatic repulsion, contributing to improved colloidal stability following centrifugation.

### 3.4. XPS Analysis of Surface Chemical States

**Figure 5:**
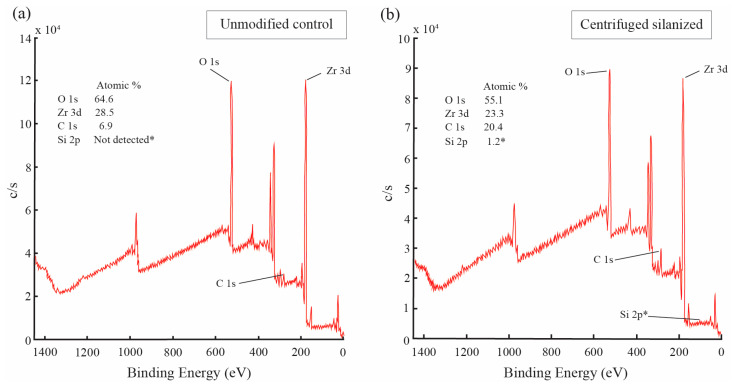
Wide-scan XPS (survey) spectra of 3Y-TZP nanoparticles before and after silanization: (**a**) unmodified control and (**b**) centrifuged silanized, showing the emergence of the Si 2p signal after surface modification. * Not detected indicates that the Si 2p signal was below the detection limit of the instrument. The detected Si 2p signal (1.2 at.%) reflects the presence of a thin organosilane layer on the nanoparticle surface.

**Figure 6:**
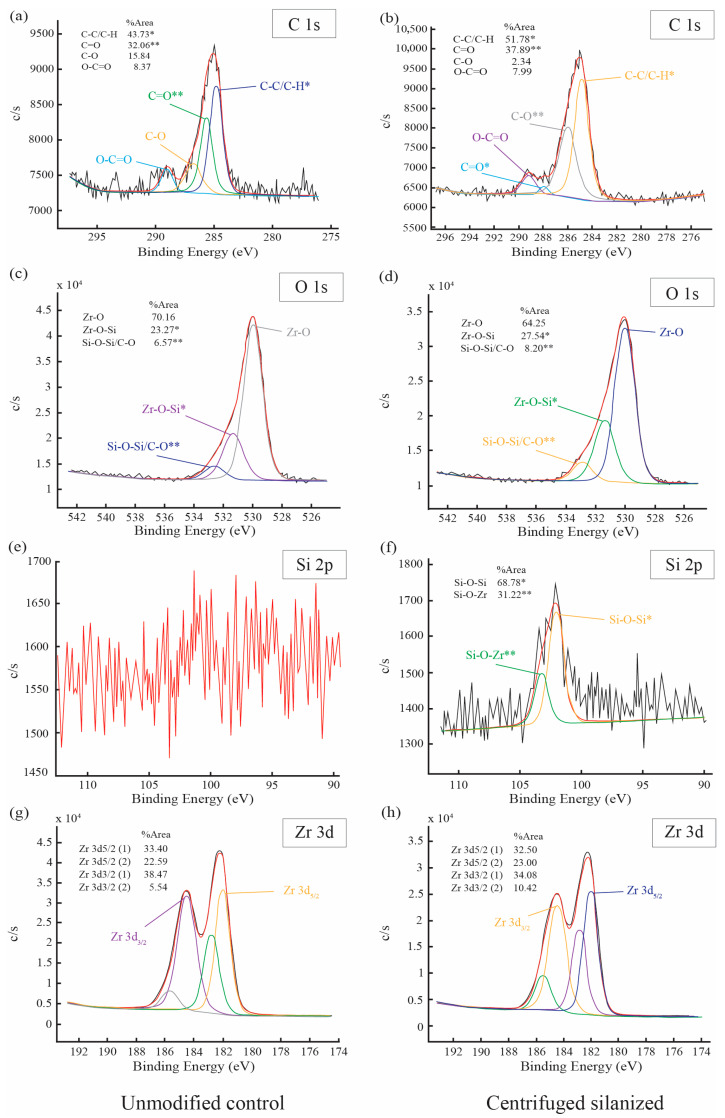
High-resolution XPS spectra of 3Y-TZP nanoparticles: (**a**,**c**,**e**,**g**) unmodified control and (**b**,**d**,**f**,**h**) centrifuged silanized sample, showing (**a**,**b**) C 1s, (**c**,**d**) O 1s, (**e**,**f**) Si 2p, and (**g**,**h**) Zr 3d regions. The black lines represent the experimental XPS spectra, while the red curves correspond to the fitted components obtained by peak deconvolution. * Indicates peaks with increased relative area (%area) after silanization, corresponding to silane-related species such as Si–O–Zr linkages. ** Indicates peaks associated with siloxane network formation (Si–O–Si), arising from condensation of silane molecules. In subfigures (**g**,**h**), the deconvoluted peaks represent the Zr 3d components corresponding to different chemical states, with individual fitted components shown as separate curves overlaid on the experimental spectra. No asterisk symbols are assigned in these regions, as no significant differences in relative peak areas were observed between the groups.

XPS spectra of the 3Y-TZP nanoparticles before and after silanization are presented in Figure 5 and Figure 6. All spectra were energy calibrated to the C 1s peak at 284.8 eV, and quantitative analysis was performed using instrument-specific sensitivity factors under identical acquisition conditions to allow relative comparison of surface compositions. Wide-scan survey spectra (Figure 5) revealed the presence of Zr and O signals in both samples. A distinct Si 2p signal was detected only after silanization. The silicon atomic percentage increased from 0.1 at.% in the control to 1.2 at.% in the silanized sample, indicating a marked relative enrichment of silicon species at the nanoparticle surface. Correspondingly, the Si/Zr atomic ratio increased from approximately 0.003 to 0.052. In parallel, the carbon atomic percentage increased from 6.9 to 20.4 at.%, consistent with the formation of an organosilane-derived interfacial layer partially attenuating the zirconia signal. The relative contributions of Zr and O decreased proportionally.

High-resolution spectra provided further insight into bonding environments (Figure 6). The C 1s spectra of both samples were dominated by the main C–C/C–H component at approximately 284.8 eV. After silanization, an increased contribution from C–O environments was observed (37.89%). Analysis of the O 1s spectra revealed a reduction in the relative lattice Zr–O component from 70.16% in the control to 64.25% after silanization, accompanied by an increase in higher binding energy oxygen species (from 23.27% to 27.54%) attributable to Si–O-related bonding environments. Deconvolution of the Si 2p spectrum in the silanized sample revealed two distinguishable components centered at 102.05 eV and 103.26 eV. Approximately 69% of the silicon species were attributed to the lower binding energy component characteristic of Si–O–Zr linkages, whereas approximately 31% corresponded to higher binding energy Si–O–Si environments associated with siloxane condensation. The predominance of the Si–O–Zr contribution suggests that a substantial fraction of surface silicon is covalently anchored to zirconia rather than present solely as condensed siloxane domains. The Zr 3d spectra of both samples displayed similar doublet positions and relative distributions, indicating that the chemical state of zirconium remained unchanged following silanization. Collectively, these findings demonstrate significant relative silicon enrichment at the nanoparticle surface and support the formation of a grafted silane interphase dominated by Si–O–Zr linkages while preserving the intrinsic zirconia lattice structure.

## 4. Discussion

The present study demonstrated that the silanization of 3Y-TZP nanoparticles significantly influenced their surface chemistry, morphology, and dispersion behavior. The combined results from FTIR and XPS confirmed successful surface modification, while SEM, TEM, and DLS analyses revealed pronounced differences in aggregation and colloidal stability between the dried and centrifuged silanized samples. Specifically, post-silanization processing played an important role in determining the effectiveness of surface modification. The centrifugation step effectively removed weakly adsorbed or oligomerized silane species, thereby enriching covalently grafted organosilane layers on the nanoparticle surface. In contrast, the dried condition retained loosely bound silane domains, which contributed to particle bridging and aggregation. This distinction defines the interfacial structure governing dispersion behavior. Combined spectroscopic, microscopic, and colloidal evidence consistently indicates that dispersion stability is governed by interfacial chemical integrity rather than by the mere presence of silane species.

FT-IR analysis confirmed the successful silanization of 3Y-TZP nanoparticles through the appearance of characteristic Si–O–Zr and Si–O–Si bands, together with methacrylate-related vibrations (C=O and C=C). These spectral features are consistent with the hydrolysis and condensation reactions of MPS on hydroxylated zirconia surfaces, indicating the formation of silane-derived surface species. The preservation of methacrylate functional groups further suggests retention of the bifunctional nature of MPS following surface attachment, which is essential for compatibility with methacrylate-based polymer matrices. These findings are in agreement with established silanization mechanisms for metal oxide substrates reported in previous studies [18,25]. However, FT-IR did not distinguish between post-silanization processing conditions, indicating that functional group detection alone does not necessarily reflect interfacial bonding integrity or dispersion stability. In contrast, surface-sensitive XPS provided more definitive mechanistic insight into the chemical state of the modified zirconia surface. The emergence of a Si 2p signal and an increase in silicon atomic percentage confirmed successful surface modification, while its predominant retention in the centrifuged sample indicates that silicon species persist primarily when covalently anchored to the zirconia surface. This suggests that centrifugation effectively removes weakly adsorbed or oligomerized silane species, whereas such loosely bound domains remain in the dried sample. High-resolution XPS spectra further demonstrated that silanization did not alter the zirconia lattice, as evidenced by comparable Zr 3d doublet positions without discernible binding energy shifts, indicating that surface modification was confined to the outermost region. In contrast, distinct differences were observed in the O 1s and C 1s regions. The silanized sample exhibited increased contributions from oxygen species associated with Si–O–Zr and Si–O–Si bonding environments, consistent with the formation of covalently grafted silane structures on the nanoparticle surface. Similarly, the C 1s spectra showed additional higher binding energy components attributable to methacrylate-related C–O and O–C=O species, reflecting the presence of an organic silane layer. Importantly, the persistence of the Si 2p signal in the centrifuged sample, together with the enrichment of Si–O-related bonding environments, confirms the formation of a chemically grafted organosilane interphase. In contrast, the dried condition promotes the retention of less stable, loosely bound silane domains, which may facilitate particle bridging and aggregation. Collectively, these findings demonstrate that post-silanization processing—particularly centrifugation—plays a critical role in stabilizing a covalently bound, chemically homogeneous interface, whereas drying leads to inferior interfacial stability and ultimately governs the observed differences in dispersion.

Morphological observations by FE-SEM and TEM, together with EDS analysis, corroborated the spectroscopic findings. Untreated 3Y-TZP nanoparticles were compact and dense, driven by strong interparticle interactions such as hydrogen bonding and van der Waals forces between hydroxylated surfaces. The occasional halo-like contrast occasionally observed around control particles is attributed to hydration layers rather than an organic coating. EDS analysis confirmed the presence of Zr and O as the dominant elements in all groups, while Si was detected only after silanization, consistent with surface modification. Importantly, comparison with the corresponding scale bars confirmed that the primary particle size remained within the nanoscale range across all groups, indicating that the observed structural differences arise from aggregation. Following silanization, distinct differences emerged depending on post-treatment processing. The dried silanized group exhibited irregular, fused-like agglomerates, suggesting interparticle bridging mediated by excess or weakly bound silane species. In contrast, the centrifuged silanized group displayed smaller, more uniformly distributed aggregates with clearly defined particle boundaries. TEM analysis further revealed a more continuous and sharply defined halo-like interfacial layer surrounding individual particles after centrifugation, consistent with the formation of a stable, covalently grafted silane layer. These morphological features directly support the XPS findings, which indicate that centrifugation effectively removes weakly adsorbed or oligomerized species while preserving covalently anchored Si–O–Zr linkages. As a result, centrifugation converts a heterogeneous adsorption layer into a chemically stable and homogeneous interphase. In contrast, drying retains loosely bound silane domains that promote particle bridging and aggregation, ultimately leading to inferior interfacial stability.

Colloidal characterization provided functional evidence linking surface chemistry to dispersion behavior. It should be noted that the Z-average obtained from DLS represents the hydrodynamic diameter of particle aggregates in suspension rather than the primary particle size. Therefore, the measured values do not reflect the intrinsic nanoscale dimensions of individual 3Y-TZP nanoparticles. Consistent with FE-SEM and TEM observations, the primary particle size in all groups remained within the nanoscale range, and the differences observed in DLS measurements arise primarily from variations in aggregation behavior. Although the control group exhibited a smaller apparent hydrodynamic diameter (225.8 ± 6.7 nm), this reflected compact inorganic aggregation rather than superior dispersion. This interpretation is supported by its relatively narrow distribution (PDI: 0.238 ± 0.012), suggesting that densely packed agglomerates behaved as single scattering entities in suspension. In contrast, the dried silanized group exhibited severe aggregation, as evidenced by a marked increase in hydrodynamic diameter to 1926.0 ± 191.8 nm, together with a broader size distribution (PDI: 0.431 ± 0.047), despite a moderately high zeta potential (+31.0 ± 0.8 mV). This observation indicates that electrostatic stabilization alone was insufficient when excess or weakly bound silane species remained on the particle surface, where they likely promoted interparticle bridging and flocculation. The centrifuged silanized group, by comparison, showed a substantially reduced hydrodynamic diameter (814.0 ± 35.0 nm) relative to the dried group, while exhibiting the highest zeta potential magnitude (+42.3 ± 0.1 mV). Although its PDI remained relatively high (0.444 ± 0.045), the reduction in Z-average together with the increased zeta potential suggests partial recovery of dispersion and improved colloidal stability after the removal of non-reactive or weakly adsorbed silane species [18]. This behavior is consistent with the formation of a more chemically homogeneous interphase, as supported by XPS and morphological observations. No significant qualitative changes in DLS distribution were observed within the measurement timeframe under the tested conditions.

From a colloid perspective, loosely adsorbed or oligomerized silane species act as heterogeneous boundary layers that promote bridging rather than stabilization. Their removal by centrifugation produces a more chemically defined interphase with more uniform surface charge characteristics. Therefore, the improved colloidal behavior of the centrifuged sample is attributed not merely to increased surface charge magnitude but to enhanced interfacial chemical uniformity and the suppression of interparticle bridging.

Taken together, the combined spectroscopic, morphological, and colloidal analyses consistently demonstrate that post-silanization processing plays an important role in defining the interfacial structure of 3Y-TZP nanoparticles. While FT-IR confirmed the presence of silane-derived functional groups in both dried and centrifuged samples, it did not differentiate the nature or stability of the interfacial bonding. In contrast, XPS provided surface-sensitive evidence of successful surface modification, as indicated by the emergence of a Si 2p signal and increased silicon atomic percentage in the centrifuged sample compared to the control. High-resolution spectra further confirmed that silicon species were predominantly present in covalently bonded environments (Si–O–Zr and Si–O–Si), consistent with the formation of a chemically grafted silane interphase. These chemical distinctions were directly supported by morphological observations, where the centrifuged group exhibited smaller, more uniformly distributed aggregates with clearly defined particle boundaries and a continuous interfacial layer, whereas the dried group showed irregular, fused agglomerates associated with interparticle bridging. Correspondingly, colloidal measurements revealed that the centrifuged sample achieved reduced hydrodynamic size and higher zeta potential, reflecting improved dispersion stability, while the dried sample exhibited severe aggregation despite comparable surface charge magnitude. Collectively, these findings indicate that centrifugation does not merely reduce excess silane but transforms a heterogeneous adsorption layer into a chemically grafted, homogeneous interphase that governs nanoparticle dispersion behavior.

From a materials science perspective, these findings establish post-silanization processing as a key design variable in the preparation of zirconia-based nanofillers for dental photopolymer systems. Achieving a chemically defined and dispersion-favorable nanoparticle surface provides a fundamental physicochemical basis for subsequent incorporation into resin-based nanocomposites. Although downstream mechanical and optical properties were beyond the scope of this study, the mechanistic insights presented here clarify how interfacial chemistry governs nanoparticle dispersion, thereby enabling more rational design of advanced dental nanocomposite materials. Building on these materials science insights, their implications for dental photopolymer systems can be further considered.

In methacrylate-based dental photopolymer resins, the silane interphase acts as the critical load transfer region between the zirconia nanofillers and the polymer network. Weakly adsorbed or oligomerized silane domains form compliant and mechanically unstable interphases that may facilitate interfacial debonding and microcrack initiation under stress. In contrast, a chemically grafted silane layer—such as that achieved after centrifugation—enables covalent coupling with the resin matrix, which is expected to enhance stress transfer efficiency and interfacial integrity [18,25]. The present findings therefore suggest that post-silanization processing, particularly centrifugation, is a key factor influencing interfacial quality in additively manufactured dental photopolymer systems.

Overall, the effective silanization of oxide nanoparticles should be evaluated based on interphase chemical definition rather than merely the presence of silane functional groups. Post-silanization centrifugation acts as an interfacial selection step that promotes the formation of a chemically coherent grafted interface governing nanoparticle dispersion behavior. By distinguishing between weakly adsorbed and covalently bonded silane species, this process defines the chemical identity of the interphase, which ultimately influences nanoparticle–polymer compatibility. This concept may be broadly applicable to other metal oxide nanofillers used in polymer-based nanocomposites.

Despite these insights, several limitations should be acknowledged. The evaluations were conducted in suspension systems using a specific silane coupling agent and thus may not fully represent behavior in complex polymer matrices or under different physicochemical environments. In addition, the characterization primarily relied on indirect assessment of nanoparticle dispersion rather than direct nanoscale quantification of primary particle size in situ. Furthermore, the absence of direct molecular-level characterization (e.g., quantitative analysis of silane species in the supernatant phase) represents a limitation of this study and warrants further investigation. Future studies incorporating a broader range of chemical conditions and advanced nanoscale characterization techniques would further validate these findings and extend their applicability, particularly in dental photopolymer systems.

## 5. Conclusions

Post-silanization processing significantly influenced the surface chemistry and dispersion behavior of 3Y-TZP nanoparticles. Centrifugation selectively removed weakly adsorbed and oligomerized silane species, resulting in a chemically grafted and homogeneous organosilane interphase dominated by Si–O–Zr bonding. This refined interphase improved dispersion stability, as evidenced by increased zeta potential, and reduced aggregation compared with direct drying. These findings demonstrate that nanoparticle dispersion is governed by interfacial chemical definition rather than the mere presence of silane functional groups. Accordingly, post-silanization centrifugation serves as a critical step in establishing a chemically coherent interphase that dictates dispersion behavior and interfacial quality. This approach provides a useful strategy for designing oxide nanofillers for polymer-based nanocomposites, including dental photopolymer systems.

## Figures and Tables

**Table 1:** Dynamic light scattering (DLS) and zeta potential analysis of 3Y-TZP nanoparticle suspensions across different surface modification conditions.

Sample Group	Z-Average (nm)	PDI	Zeta Potential (mV)
Unmodified control	225.8 ± 6.7 ^a^	0.238 ± 0.012 ^a^	+33.4 ± 0.4 ^a^
Dried silanized	1926.0 ± 191.8 ^b^	0.431 ± 0.047 ^b^	+31.0 ± 0.8 ^a^
Centrifuged silanized	814.0 ± 35.0 ^c^	0.444 ± 0.045 ^b^	+42.3 ± 0.1 ^b^

Values are expressed as mean ± standard deviation (SD) from triplicate measurements (*n* = 3). Different superscript letters within the same column indicate statistically significant differences between groups (*p* < 0.05), as determined by one-way ANOVA followed by Tukey’s post hoc test.

## Data Availability

All relevant data are included within the article.
